# Multimodal Analgesia in the Perioperative Period of Major Surgeries: An In-depth Analysis

**Published:** 2025-09-08

**Authors:** Sean Kincaid, Justine How, Devendra K Agrawal

**Affiliations:** Department of Translational Research, College of Osteopathic Medicine of the Pacific, Western University of Health Sciences, Pomona, California, USA

**Keywords:** Anesthesia, Chronic post-surgical pain, ERAS protocol, Multimodal analgesia, Nociception, Pain management, Surgery

## Abstract

The successful management of postoperative pain remains a significant challenge to patient recovery following high-risk surgeries, often leading to the overuse of opioids and increasing dangers for developing chronic post-surgical pain (CPSP). CPSP is defined as pain persisting for at least 3 months after surgery, beyond the expected healing window. CPSP can develop after any type of surgery, but especially very traumatic ones- where nerve injury, inflammation, and abnormal central sensitization cause acute postoperative pain to transition into chronic pain. Multimodal analgesia (MMA) is an integrated pain management approach that employs a wide range of drug interventions with the end goal of achieving a synergistic effect in pain reduction and recovery. This method is used to reduce the necessity for opioids due to their addictive properties and other detrimental side effects. Anchored in Enhanced Recovery After Surgery (ERAS) protocols, MMA is a tailored approach that takes into consideration various pain pathways, such as nociceptive, neuropathic, and inflammatory. These methods can widely differ across various surgical categories, as each patient and procedure present distinct complications to address. Current studies offer a vast array of interventions with shifting impacts on recovery, though there is general agreement on certain specific, consistently effective approaches. This review critically reviewed the most widely accepted MMA strategies across orthopedic, thoracic, abdominal, breast, and amputation procedures, while also identifying areas for further optimization. Overall, the multimodal analgesia reduces opioid intake in the postoperative setting and benefits patients undergoing multiple procedures. However, there is a need for integrative, patient-tailored algorithms supported by predictive analytics and perioperative data to personalize MMA plans. Further investigations using high-quality, procedure-specific randomized controlled trials are warranted to evaluate short-term analgesic success and long-term quality-of-life metrics.

## Introduction

**Figure 1:**
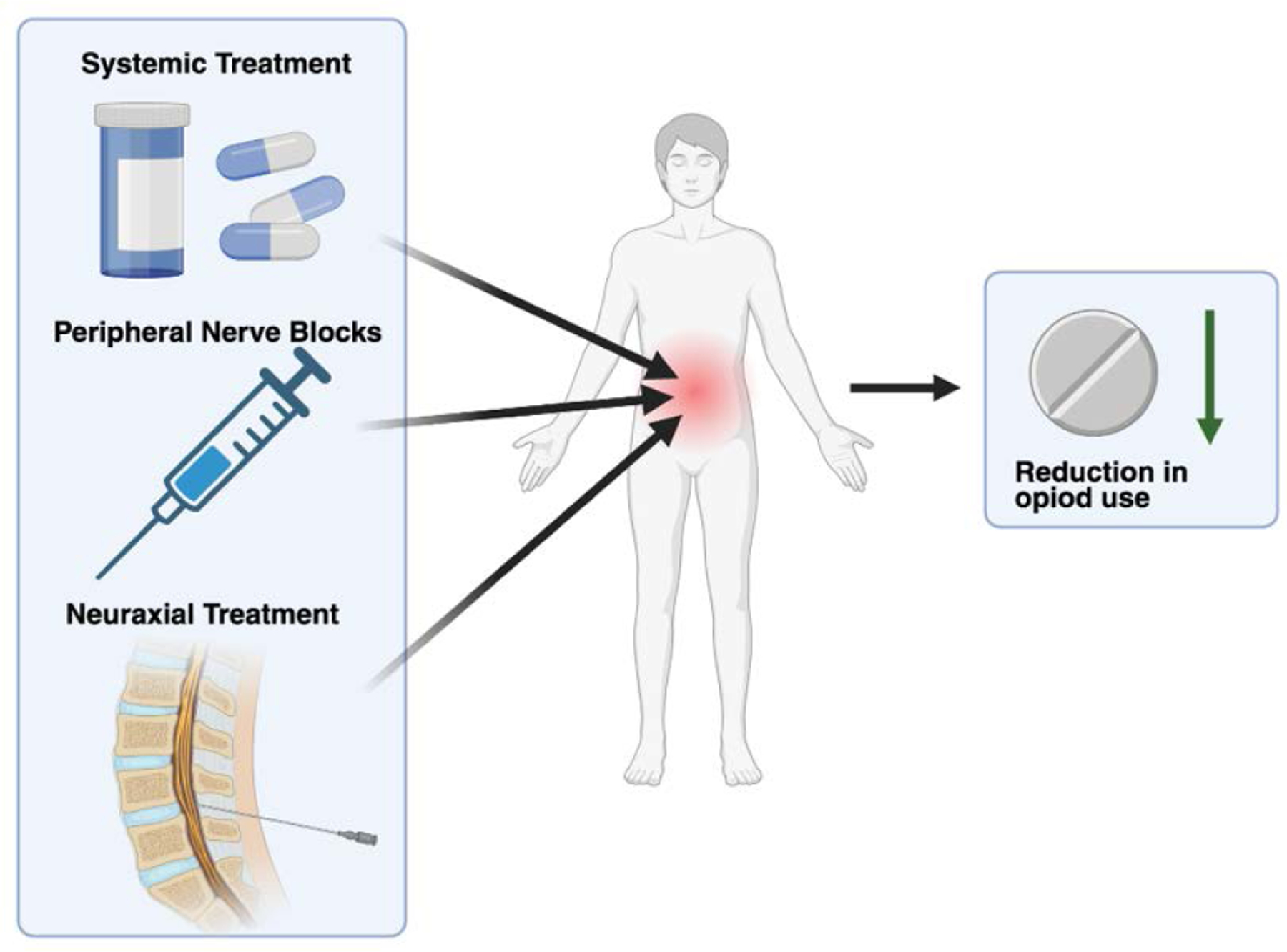
Multiple drug pathways work together to reduce patient pain levels while also reducing opioid intake.

Multimodal analgesia (MMA) is a synergistic, combined approach to postoperative pain management. The combination of medications and analgesic techniques in MMA targets multiple facets of the neurochemical pain pathway, resulting in a greater overall reduction in pain. The basic components of these analgesic regimens will contain neuraxial treatments, peripheral nerve blocks, local infiltration, and systemic treatment (Figure 1). Common systemic treatment will typically include nonsteroidal anti-inflammatory drugs (NSAIDs), acetaminophen, and gabapentinoids as these drugs are rapidly absorbed and distributed throughout the body [1].

The increasing need for MMA comes in the face of the ever-growing opioid epidemic. Current data shows the deaths from prescription opioid-related deaths have jumped from 4,030 in 1999 to 32,445 in 2016, representing an 8-fold increase [2]. Like all addictive drugs, opioids act directly on the mesocorticolimbic dopamine system, leading to reinforcement of drug-seeking behaviors [3]. These qualities, in combination with opioid reliance for pain reduction after surgery, have led to significant morbidity and mortality among patient populations [4]. This concern surrounding the dangers of opioid prescription has led doctors to find safer alternative approaches to pain management through the implementation of MMA regimens (Figure 1). Although opioids are still used in many MMA treatment plans the overall dependency on them is substantially reduced [5].

The success of MMA has led it to become a valuable component of the Enhanced Recovery After Surgery (ERAS) protocols, attributed to its reduction in length of stay after surgery and decreased need for opioid use [5]. ERAS protocols are multimodal perioperative care pathways to maintain pre-operative organ function and reduce the pain and stress responses that come postoperatively [6]. The ERAS society guidelines aim to reduce complication rates, but many full outlines are still under development [7]. This illustrates the purpose of this review in compiling the most up-to-date and understood MMA methods among major surgical procedures.

There is a wide range of surgical procedures that can lead to both acute and chronic postoperative pain (CPSP), and each of them comes with different demands in terms of MMA. Approximately 75% of patients experience pain after surgery, and of these patients, 86% rated it as moderate, severe, or extreme [8]. Studies show that the leading procedures affected by postoperative pain include orthopedic, thoracic, cardiac, abdominal, breast, and amputation surgeries [9,10]. With the variety of surgical procedures and pain pathways involved, it is important to understand the most current and supported approaches to MMA in these procedures, and this review aims to highlight these findings in depth.

## Mechanisms of Pain and Recovery in Post-Surgical Patients

**Figure 2:**
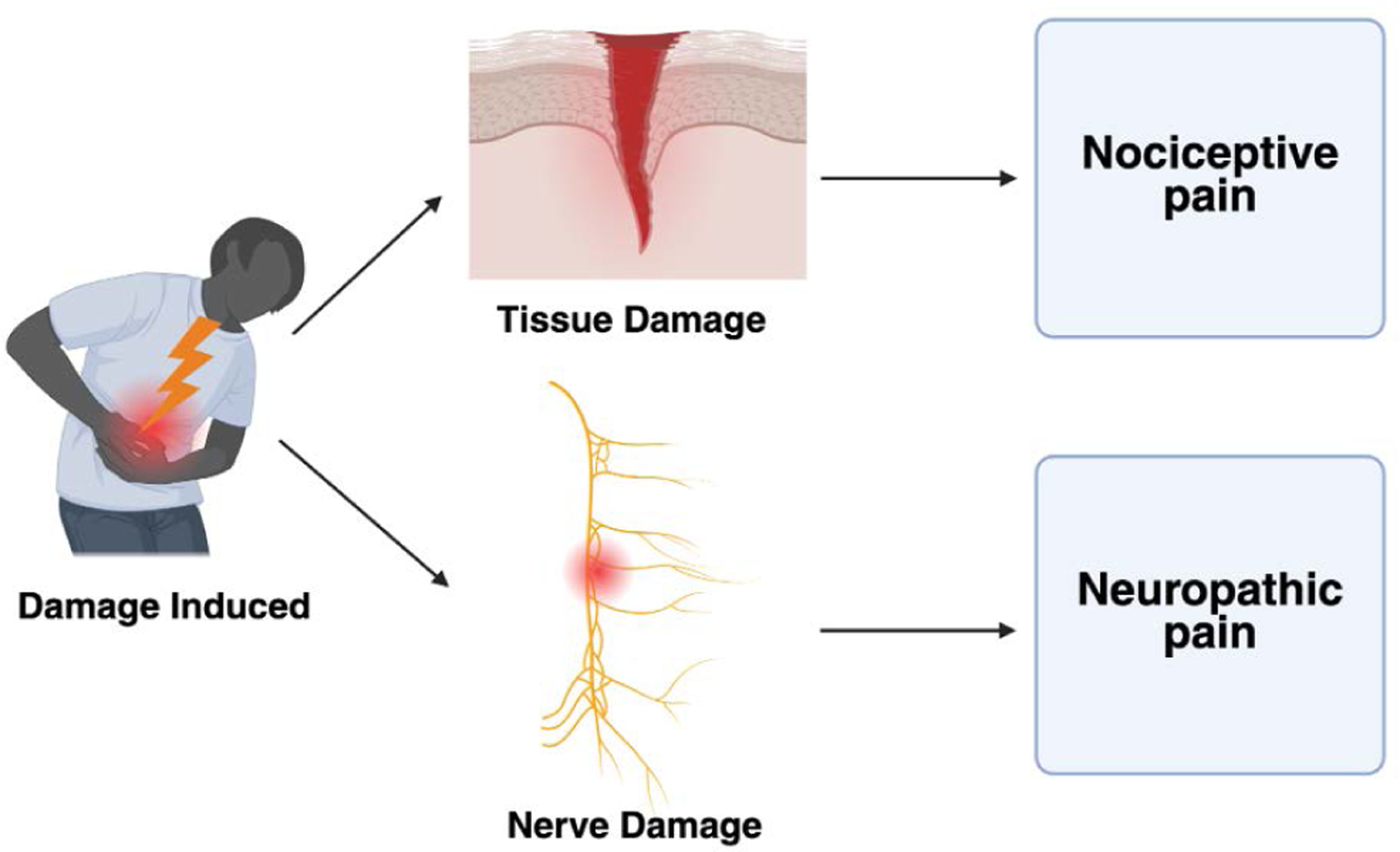
Damage during surgery can affect either tissue or nerves, resulting in nociceptive pain and neuropathic pain, respectively.

In order to better understand the role of MMA, the experience of pain itself must first be investigated. In general, pain is classified as either neuropathic or nociceptive, with each containing its respective subclasses [11]. Nociceptive pain can be described as a detection of tissue damage by specialized transducers that are attached to A, delta, and C fibers. These transducers can be influenced by both inflammatory and neural changes within their immediate environment [12]. The primary compounds used to reduce nociceptive pain include NSAIDs, corticosteroids, valued for their anti-inflammatory properties, and acetaminophen [13]. As a result, these agents are widely incorporated into standard postoperative pain management protocols. Neuropathic pain, while slightly less common than nociceptive pain, is another highly prevalent pathway targeted by MMA. Neuropathic pain is widely defined as pain caused by a lesion or disease of the somatosensory system (Figure 2). Damage to this system can lead to altered transmission of sensory signals, which can result in conditions like neuralgia, radiculopathy, and peripheral nerve pain [14]. Specific analgesic approaches look to target these pathways using gabapentinoids, SSRIs, nerve blocks, and local anesthetics such as lidocaine [15,16].

Together, these classifications and their corresponding treatment modalities form the foundation of MMA, and they must be tailored specifically to each procedure in order to optimize patient outcomes.

## Multimodal Strategies for Specific Surgical Recoveries

### Orthopedic

Orthopedic surgery is a leading cause of acute and chronic postoperative pain among patient populations, making multimodal analgesia a major contributor to its treatment. Acute pain following orthopedic surgery is typically the result of mechanically induced damage to tissue integrity. The large amount of tissue damage at the surgical site leads to massive local inflammatory responses and, if left untreated, can modulate pain perception to become hypersensitive in the peripheral sensory neurons. This hypersensitivity can be long-lasting and results in CPSP. For these reasons, inflammatory mediators that sensitize nociceptors, such as prostaglandins and neurotransmitters, are the primary targets of drug therapies [17].

To achieve a reduction in inflammation, the consensus approach across surgical fields is the use of NSAIDs and Selective COX-2 inhibitors [18–20]. These COX-2 inhibitors are specifically designed to target and reduce the inflammatory pathway. Among spinal surgeries, a meta-analysis found improved pain scores and reduced opioid intake with the use of adjunctive NSAIDs across 17 studies [18]. In a separate analysis on knee and hip arthroplasties, patients who received COX-2 inhibitors reported significantly lower pain scores with similar side-effect profiles against placebos [19]. However, there is contradictory evidence that suggests the potential for detrimental effects with the use of NSAIDs in the setting of tendon and ligament repair, but research thus far has been inconclusive [21]. Overall, this demonstrates the importance of a tailored approach to individual patients on a case-by-case basis.

Another widely implemented method is the use of regional or local anesthetics during the course of an orthopedic procedure. There is a wide range of anesthetics, such as lidocaine or bupivacaine, that are used to block local pain receptors around surgical wounds. Both lower extremity and brachial plexus blocks have demonstrated a significant decrease in postoperative opioid consumption and in overall pain scores [5]. In a related technique known as local infiltration a study using the injection of a ropivacaine mixture into the surgical site yielded satisfactory pain control with no need for additional morphine in two-thirds of knee and hip replacement patients [22].

**Figure 3:**
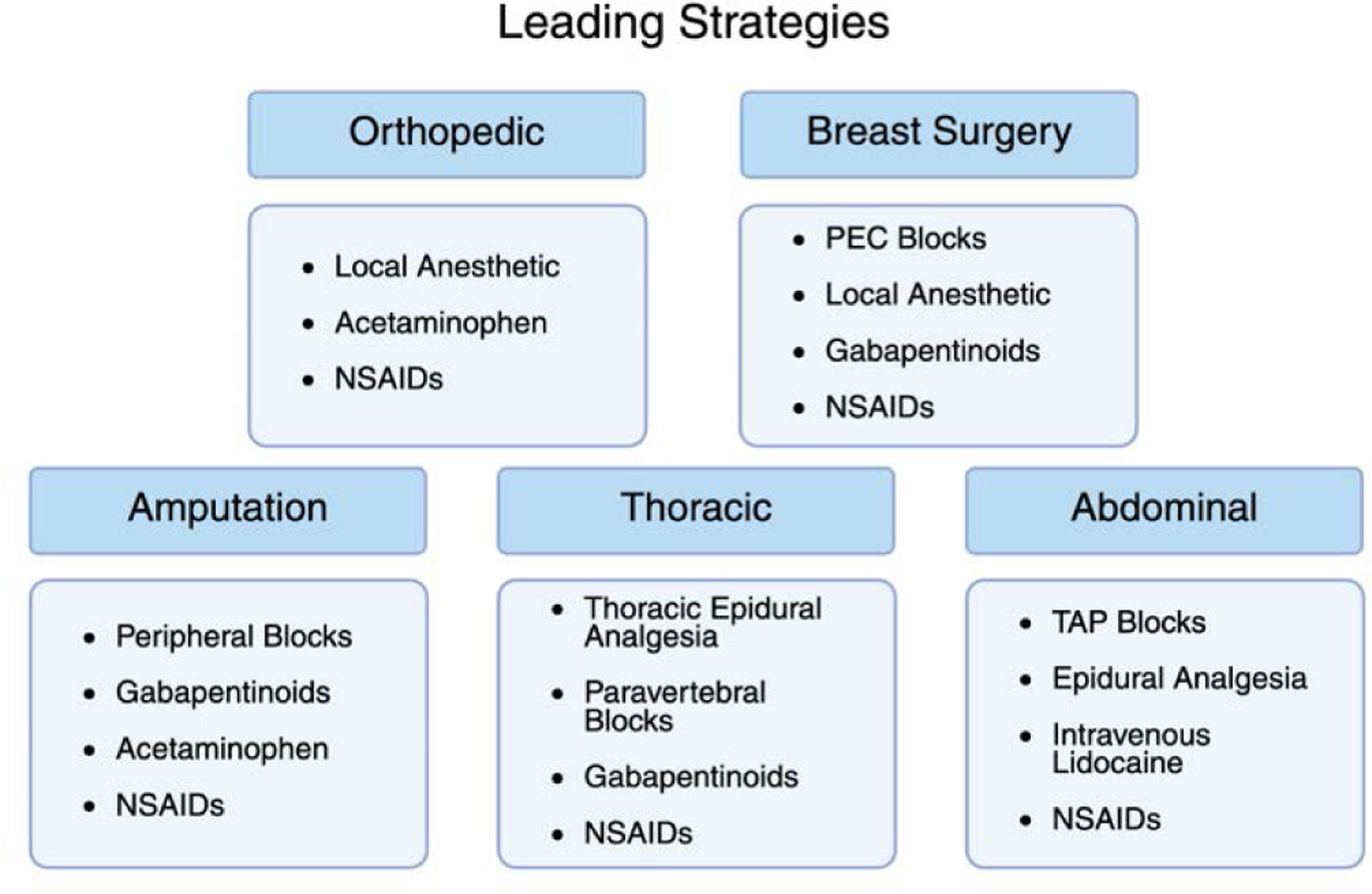
The leading strategies for MMA vary greatly between each individual surgical procedure.

In combination with previously mentioned methods, acetaminophen is a critical contributor to MMA in orthopedics. Acetaminophen has been utilized for its effective analgesic and antipyretic properties for over a century. It has been approved for use in the US for more than 60 years due to its relative safety as a drug [23]. This makes it an excellent addition to MMA following most operations, as it can mitigate the need for opioids with few side effects. Orthopedic spine studies have shown a greater reduction in opioid consumption with acetaminophen in combination with NSAIDs [24]. This symbiosis of drugs is the cornerstone to achieving high success rates in orthopedic MMA, and the three-pronged approach of NSAIDS, acetaminophen, and local/regional anesthesia should be utilized together for optimal patient recovery (Figure 3).

### Breast Surgery

Postoperative pain management after breast surgery can be complex, secondary to the vast innervation of the chest wall and the frequent involvement of both somatic and neuropathic pain components. Pain following breast procedures-including lumpectomy, mastectomy, and reconstruction- can be substantial, and if inadequately controlled, may progress to CPSP (like any traumatic procedure). To mitigate this, MMA plays a critical role in achieving both effective pain control and opioid minimization in the post-surgical period.

A mainstay of MMA in breast surgery is the use of NSAIDs and selective COX-2 inhibitors, which target prostaglandin synthesis and reduce inflammatory sensitization of nociceptors. These medications not only lower pain intensity, but have also been shown to decrease opioid use when used as adjuncts in perioperative care [25]. Though they carry risks such as bleeding or renal concerns, evidence supports their use when patient-specific contraindications are considered [26]. Acetaminophen further contributes to MMA protocols due to its strong safety profile and additive analgesic effects when used alongside NSAIDs. As a centrally acting agent, it effectively reduces postoperative pain and has been included in enhanced recovery protocols for its opioid-sparing benefit, especially when administered on a scheduled basis [27,35]. In addition, gabapentinoids such as gabapentin and pregabalin are commonly administered preoperatively or postoperatively in breast surgery due to their efficacy in addressing neuropathic pain. Multiple studies have demonstrated their effectiveness in lowering both acute pain scores and opioid consumption in breast surgery patients [28,66,67].

A significant advancement in regional anesthesia for breast surgery is the pectoralis nerve block (PECS I and II). These ultrasound-guided blocks anesthetize the medial and lateral pectoral nerves as well as the intercostobrachial and thoracic intercostal nerves, significantly reducing intraoperative and postoperative pain. PECS blocks have rapidly gained popularity for their simplicity and strong efficacy, with studies showing decreased use of opioids and improved pain control compared to general anesthesia alone [29]. In more extensive procedures or when deeper analgesia is needed, paravertebral blocks (PVBs) can be utilized. PVBs provide unilateral analgesia along multiple thoracic dermatomes and are associated with improved postoperative recovery and patient satisfaction [30,31].

Local anesthetic infiltration is another supportive technique, where agents like bupivacaine or ropivacaine are injected directly into the surgical field. This provides immediate analgesia in the peri-incisional area. Combining local infiltration with systemic and regional techniques allows for the enhancement of overall pain control without increasing side effect burden [32,33].

MMA protocols in breast surgery rely on complementary combinations of systemic medications and regional techniques to achieve optimal outcomes. The layered approach (using anti-inflammatory agents, centrally acting drugs, nerve modulators, and targeted blocks) not only improves pain scores but has been consistently shown to reduce opioid reliance (Figure 3). As evidence continues to evolve, the integration of personalized MMA in breast surgery will remain central to enhanced recovery and quality perioperative care.

### Amputation

Amputations present a very high risk for both severe acute pain and the development of chronic pain, including phantom limb pain (PLP). PLP is defined as the sensation of pain that feels like it’s coming from a limb (or even a missing tooth, eye, or breast) that is no longer present [34]. Trauma to peripheral nerves and surrounding tissues can lead to a heightened nociceptive and neuropathic pain response, which can become difficult to manage with opioids alone [34,35]. For this reason, MMA has become a significantly important part of pain control in amputations, aiming to address the diverse mechanisms of pain transmission and reduce the long-term burden of chronic pain.

Unsurprisingly, NSAIDs are frequently used in MMA regimens, including amputations. These medications function by inhibiting prostaglandin synthesis and suppressing the inflammatory response caused by extensive tissue and nerve damage [36]. In amputations, NSAIDs are mainly used for postop pain, and do not prevent or alleviate PLP [35]. A 2006 review emphasized the role of selective COX-2 inhibitors in minimizing postop opioid use while maintaining effective pain control [37]. However, as mentioned prior, caution is warranted with these medications in patients with compromised renal function. Acetaminophen also serves as a well-tolerated and effective adjunct to NSAIDs and regional techniques, with few side effects- but caution should be taken in patients with liver dysfunction. When given around-the-clock in the immediate postop period, it enhances analgesic coverage while limiting the need for opioids [35,41].

Gabapentinoids, including gabapentin, play a critical role in addressing the neuropathic component of post-amputation surgery pain. A double-blind placebo-controlled trial found gabapentin effective in reducing both acute pain and PLP following amputation.^40^ These agents act centrally to inhibit calcium channel activity on hyperactive neurons, reducing the likelihood of central sensitization and CPSP [40,68].

One of the most impactful modalities of MMA in amputations is regional anesthesia, particularly peripheral nerve blocks. Techniques such as femoral, sciatic, or adductor canal blocks can provide complete surgical anesthesia and prolonged postoperative analgesia in both below- and above- knee amputations. During the COVID-19 pandemic, studies demonstrated that regional techniques alone could provide sufficient surgical anesthesia- avoiding the need for general anesthesia in high-risk patients.^38^ Beyond intraoperative use, perineural catheters can be employed to prolong analgesia into the post-op period, significantly reducing opioid use [39].

The concept of preemptive analgesia (administering analgesics before tissue injury) is of note in amputation cases. Studies have shown that preemptive analgesia strategies, like regional blocks or early gabapentinoid use, can attenuate central sensitization and lower the incidence of persistent pain syndromes [35,42–45].

The most successful MMA protocols for amputation surgery integrate multiple layers of pain control: anti-inflammatory agents, regional nerve blocks, central neuromodulators, and non-opioid analgesics (Figure 3). This kind of comprehensive approach not only improves immediate postoperative comfort but is essential in reducing the transition to chronic and phantom limb pain. Given the complex pain physiology in this patient population, individualized regimens and close follow up are vital components of postoperative care.

### Thoracic Surgery

Thoracic surgery is associated with significant postoperative pain, and because of its impact on respiration, inadequate pain management can often lead to serious complications for the patient. The disruption of normal breathing mechanics can lead to restrictive ventilation patterns, decreased compliance, and a reduction in functional residual capacity. High levels of post-surgical pain amplify these effects and impair the patient’s ability to achieve regular tidal volume. This impairment can then result in life-threatening complications such as hypoxemia, hypercarbia, pneumonia, and the eventual need for mechanical ventilation. These risks emphasize the critical importance of a properly managed pain protocol following thoracic procedures.

The primary operations in thoracic surgery are the thoracotomy and video-assisted thoracic surgery (VATS). Both procedures carry a high risk for nerve injury, muscle damage, pleural disruption, and inflammation. To address these challenges, the leading strategies involve the use of non-opioid systemic analgesics and regional anesthesia such as thoracic epidural analgesia (TEA) and paravertebral blocks (PVB) [46].

Thoracic epidural analgesia is the most commonly used modality among thoracic surgery patients and is widely considered to be the gold standard of treatment [47]. TEA is administered through the insertion of an epidural catheter targeting the thoracic nerve segments, followed by the infusion of local anesthetics. The results of this treatment include improved pain control, reduced pulmonary complications, earlier extubation, and fewer cardiac dysrhythmias. In over 4000 patients, the complication rate was 3.1% indicating a high degree of patient safety. Furthermore, studies demonstrated superior levels of pain control when compared against parenteral opioids [48]. The overall analgesic effects combined with their improvement to pulmonary function have led to TEA becoming a highly recommended approach in thoracic MMA [49].

Another common approach to regional anesthesia is the paravertebral block. While similar in effect to TEA, there is an ongoing dispute over the risks and benefits of each treatment. Paravertebral blocks involve the injection of a local anesthetic into the region lateral to the spinal nerves, allowing for an analgesic effect across several dermatomes from a single injection site. The debate between TEA and PVB has shown varying evidence on both sides with significant heterogeneity, indicating a potential area for future research [50]. In support of PVB, one study concluded that PVB was equally as effective as TEA with fewer side effects [51]. However, overall pain scores are relatively consistent between the two approaches [50].

The use of systemic analgesics remains common across many MMA protocols, with thoracic procedures focusing on NSAIDs and Gabapentinoids. NSAIDs have been encouraged among standardized approaches for thoracotomies. Selective NSAIDs may be preferred as they have been shown to yield less adverse effects regarding surgical bleeding and renal dysfunction [52]. Gabapentinoids have been recommended for perioperative usage to reduce thoracic neuropathic pain. Meta-analysis indicates a significant reduction in pain scores at 0 hours, pain scores at 24 hours, and overall neuropathic pain [53]. In combination with regional anesthetic approaches, systemic analgesics work to create a complete and effective MMA treatment outline for thoracic procedures (Figure 3).

### Abdominal Surgery

Major abdominal surgery covers a wide range of procedures with broad pain and analgesic requirements. In 2010, there were 7.4 million major abdominal surgeries in the seven largest countries. The leading procedures were laparoscopy, laparotomy, and cholecystectomy. When investigating the sources of chronic post-surgical pain the abdominal, perineal, genital, and anal regions were identified as the most common contributors [54]. As such, there are many different approaches to dealing with post-surgical pain following abdominal procedures. Currently, the leading techniques include intravenous lidocaine, transversus abdominis plane (TAP) blocks, epidural analgesia, and NSAIDs [55].

Intravenous lidocaine (IVL), utilized for its benefits in reducing pain intensity, nausea, duration of ileus, and postoperative opioid requirements, has been thoroughly supported through recent research [56,57]. A meta-analysis by Marret et al. [58] found that IVL yielded a pain intensity reduction of 5.9 mm (Rated on VAS), an 8.3 h reduction in duration of ileus, and a 0.84 day reduction in length of hospital stay [58]. Levy et al. [59] saw a pain scale reduction of 1.1 for laparoscopic procedures and a 0.7 reduction for open abdominal procedures despite a decrease in opioid consumption [59]. Overall, IVL is a critical tool when selecting for abdominal MMA.

Another effective form of MMA in the abdominal region is the transversus abdominis plane (TAP) block. The TAP block is performed through the insertion of a needle through the external and internal obliques, followed by the injection of local anesthetic to effectively block the nerves of the abdominal wall. Studies show TAP blocks can reduce the need for morphine in the immediate postoperative period [60]. Combined meta-analysis has also shown TAP blocks to be safe with no significant side effects. Additionally, it is important to note that the TAP block had similar reductions in pain ratings compared against single-shot spinal anesthetic with intrathecal morphine [61]. One study did note a greater analgesic result when using epidural analgesia, so further research could be needed to determine the superior approach [62].

Optimal pain management after abdominal surgery involves the targeting of multiple pathways, with NSAIDs and acetaminophen once again playing a critical role in thorough treatment [63]. The use of NSAIDs following colorectal surgery was found to significantly improve time to gut recovery, with a focus on their anti-inflammatory effects [64]. Furthermore, a systematic review of NSAIDs compared against codeine + acetaminophen found a similar reduction in pain scores across major abdominal surgery patients [65]. In totality, the combination of these analgesics has been shown to offer optimal results among patients, and MMA once again proves its value (Figure 3).

## Challenges and Future Directions

Despite the widespread adoption of MMA in postoperative pain management, challenges remain in optimizing its implementation across diverse surgical settings. One such challenge is the lack of standardized protocols that are both evidence-based and adaptable to individual patient needs, surgical complexity, and institutional resources. The current literature shows considerable heterogeneity in analgesic and dosing regimens and strategies, and regional anesthesia techniques, of which can restrict cross-comparability and consistent application. While the opioid-sparing benefits of MMA are well documented, certain components- like the aforementioned medications and regional blocks- carry their own risks and contraindications, requiring nuanced clinical judgment that may not be uniformly practiced. There is also an underrepresentation of long-term outcomes data, particularly regarding CPSP and functional recovery- making it difficult to assess the sustained efficacy of specific MMA approaches. Future research should prioritize high-quality, procedure-specific randomized controlled trials that evaluate not only short-term analgesic success but also long-term quality-of-life or pain metrics. MMA could benefit from integrative, patient-tailored algorithms supported by predictive analytics and perioperative data to personalize pain management plans. As the field continues to evolve, collaborative efforts between anesthesiology, surgery, and pain medicine will be essential in developing unified, scalable MMA strategies that balance efficacy, safety, and accessibility [26,41,63].

## Conclusion

Throughout the current research, MMA shows a wide range of benefits for patients while reducing opioid use after surgery. As surgical care evolves and patient-centered outcomes remain a priority, MMA has become a key part of recovery protocols. It offers tailored, evidence-based strategies for various procedures. Whether in orthopedic, thoracic, abdominal, breast, or amputation surgery, there is strong evidence that MMA approaches should be personalized to each patient and surgical situation to maximize effectiveness and safety. This review highlights the most current and validated methods to support optimal postoperative protocols. Ultimately, this can improve recovery times, reduce complications, and help shift toward safer, more effective, and opioid-free perioperative care. As new techniques and pain relief options are developed, ongoing research and teamwork will be crucial in refining these strategies for optimal outcomes across different surgical groups.

## Key Points

- Multimodal anesthesia (MMA) is a synergistic pain management strategy designed to minimize opiate use and enhance post-operative pain and recovery.
- MMA is grounded in the Enhanced Recovery After Surgery (ERAS) protocols, which aim to reduce complications and improve surgical outcomes.
- Effective MMA combines systemic drugs (NSAIDs, acetaminophen, gabapentinoids) with local/regional anesthetics tailored to specific procedures.
- Orthopedic surgeries benefit from a 3-pronged MMA approach using NSAIDs, acetaminophen, and regional anesthesia, significantly reducing opioid requirements.
- In breast surgery, MMA strategies include NAIDs/COX-2 inhibitors, gabapentinoids, and regional blocks like PECS and PVBs to prevent chronic pain.
- MMA in amputation focuses on preventing severe acute and phantom limb pain using NSAIDs, nerve blocks, gabapentinoids, and pre-emptive analgesia.
- Thoracic MMA protocols emphasize epidural analgesia and PBVs, along with NSAIDs and gabapentinoids, to control pain and also present respiratory complications.
- Abdominal MMA effectively incorporates IV lidocaine, TAP blocks, epidural analgesia, and NSAIDs for quicker recovery and reduction in opiates.
- Across all surgical domains, MMA consistently reduces opiate use and improves pain outcomes, but optimal strategies vary by procedure and patient characteristics, requiring custom tailoring per case.
